# International normalized ratio testing with point-of-care coagulometer in healthy term neonates

**DOI:** 10.1186/1471-2431-14-179

**Published:** 2014-07-09

**Authors:** Shigeo Iijima, Toru Baba, Daizo Ueno, Akira Ohishi

**Affiliations:** 1 Department of Regional Neonatal-Perinatal Medicine, Hamamatsu University School of Medicine, Hamamatsu, Japan

**Keywords:** International normalized ratio, Coagulation screening, Coagulometer, Vitamin K deficiency bleeding, Neonate

## Abstract

**Background:**

Neonates routinely receive vitamin K to prevent vitamin K deficiency bleeding, which is associated with a high mortality rate and a high frequency of neurological sequelae. A coagulation screening test might be necessary to detect prophylactic failure or incomplete prophylaxis. However, venous access and the volume of blood required for such testing can be problematic. CoaguChek XS is a portable device designed to monitor prothrombin time while only drawing a small volume of blood. Although the device is used in adults and children, studies have not been performed to evaluate its clinical utility in neonates, and the reference value is unknown in this population. The objectives of the present study were to determine the reference intervals (RIs) for international normalized ratio (INR) using the CoaguChek XS by capillary puncture in healthy term neonates, to evaluate factors that correlate with INR, and to evaluate the device by assessing its ease of use in clinical practice.

**Methods:**

This study included 488 healthy term neonates born at a perinatal center between July 2012 and June 2013. The INRs determined by CoaguChek XS were measured in 4-day-old neonates.

**Results:**

The enrolled neonates were orally administered vitamin K 6-12 h after birth. A RI for INRs in 4-day-old neonates was established using the CoaguChek XS with a median value of 1.10 and a range of 0.90–1.30. A significant difference in the INR was noted between male (median value, 1.10; RI, 0.90–1.30) and female (median value, 1.10; RI, 0.90–1.24) neonates (p = 0.049). The INR was found to correlate with gestational age, birth weight, and hematocrit value.

**Conclusions:**

The CoaguChek XS device is safe, fast, and convenient for performing INR assays in neonates. Our study is the first to establish a RI for INRs that were measured using the CoaguChek XS in healthy term neonates.

## Background

Neonatal vitamin K deficiency bleeding (VKDB) causes digestive tract bleeding in the early neonatal period and intracranial hemorrhage in early infancy. The incidence of VKDB in infants without prophylaxis has been reported to range from 4.4 to 10.5 per 100,000 live births in Asia and Europe [1]. In Japan, a nation-wide survey in 1981, before vitamin K prophylaxis was recommended, revealed that 7.1 cases of VKDB occurred per 100,000 births. In 1988, to prevent this condition, a protocol of three oral doses of vitamin K, 2 mg each, was recommended for all full-term neonates; these are given on the day of birth, upon discharge from the maternity hospital, and at 1 month of age. With the incorporation of these prophylaxis guidelines, the frequency of VKDB in Japan has significantly decreased. In a nation-wide survey in 2005, the incidence of VKDB was determined to be 1.9 per 100,000 births [2]. However, prophylactic failure has continued to occur in infants who were later proven to have cholestatic liver disease, although other main risk factors include parental refusal and accidental omission [3,4]. In addition, oral vitamin K prophylaxis is considered incomplete if at least one oral dose but not all recommended doses are given according to age, or if an inadequate dose or preparation is given, although it is considered complete if all recommended doses are given at the time of bleeding. According to the latest nationwide survey in Japan, 89% of infants with VKDB had received prophylactic vitamin K at least once during or after the neonatal period [2]. In the current system, prophylactic failures or incomplete prophylaxis cannot be identified before bleeding occurs. Therefore, a coagulation screening test, which is a reliable method to detect prophylactic failures or incomplete prophylaxis, might be warranted. In neonates, however, two major difficulties arise when taking blood samples for coagulation tests: the challenge of venous access and the comparatively large amount of blood required.

Portable point-of-care (POC) analytical instruments for measurement of capillary whole blood prothrombin time (PT), which is expressed by the international normalized ratio (INR), have been available for the last decade, and the system offers convenient and accurate anticoagulant monitoring in adult patients [5]. Using this method can reduce the volume of blood needed for tests, and capillary blood samples from heel pricks may provide a solution to difficult venous access. To date, however, only a few studies evaluating such portable coagulometers have been conducted in children [6-8], and no studies in neonates.

The main objectives of the present study were to establish reference intervals (RIs) of POC capillary puncture INR for a coagulation screening test in healthy term neonates and to evaluate factors that correlate with INR. In addition, we evaluated the validity and utility of the CoaguChek XS device in neonates.

## Methods

### Patients and data collection

The subjects were healthy neonates born at Hamamatsu University Hospital from July 1, 2012 to June 30, 2013. They were born at full term (37–41 weeks of gestation) with normal birth weight (2500–3999 g), and all of them were given 2 mg of vitamin K syrup (Menatetrenone Kaytwo Syrup, Eisai Co., Ltd., Tokyo, Japan) orally 6–12 h after birth. Our intention was to evaluate the RI of INR in healthy term neonates and, therefore, all neonates hospitalized in the neonatal intensive care unit (NICU) were excluded from this portion of the study. After informed consent was obtained from the parents, neonates were enrolled prospectively in this study.

A capillary whole blood sample was obtained by heel prick at the same time of a blood sampling for the screening of inherited metabolic disorders (mass-screening) and the routine serum bilirubin and hematocrit measurement when the babies had a health checkup at 4 days after birth. The coagulation screening test consisted of PT using a portable coagulometer, CoaguChek XS, and the INR was calculated. A single heel prick was performed and the first drop of capillary blood (at least 8 μL) obtained was then applied to the test strip, which was already inserted into the CoaguChek XS device. Next, a whole blood sample (approximately 40 μL) was collected into a heparinized capillary tube for measurement of serum bilirubin and hematocrit values. Finally, another whole blood sample (at most 200 μL) for mass-screening was collected on the filter paper. Blood samples were drawn by four dedicated and experienced NICU doctors who had received training in the use of the CoaguChek XS. The serum bilirubin level was measured by the optical density method using Bilmeter F (Mochida-Siemens, Tokyo, Japan). Hematocrit was measured by the microhematocrit method. This study was reviewed and approved by the Hamamatsu University School of Medicine Ethics Committee.

### Portable coagulometer

CoaguChek XS is a small, battery-powered, handheld meter that is portable and efficient. It measures the INR using whole blood obtained by capillary puncture. The procedure involves insertion of a test strip into the monitor and application of a drop of blood (8 μL) onto the test strip. The monitor uses an electrochemical method to determine the PT after activation of coagulation with a recombinant human thromboplastin within the test strip. The mean international sensitivity index (ISI) for the CoaguChek XS PT test is 1.01. The PT is then converted to an INR using the ISI that was previously determined and encoded on the chip for each lot of test strips. The INR result is usually provided in less than 1 minute (approximately 10 seconds after application of blood to the test strip).

### Assessment of utility

Instances in which a PT result could not be obtained were recorded and the failure rate of the method was calculated. In addition, the doctors were asked questions about the utility and ease of use of the CoaguChek XS system.

### Determination of accuracy

Using linear regression analysis, the accuracy of CoaguChek XS was assessed by comparing INRs obtained using this method to those measured using the laboratory gold standard method in neonates who were admitted to the NICU over the same period. The patients underwent a coagulation study including determination of INR according to their clinical indications, for example, pre-and postoperative major surgery and other causes of bleeding. Blood samples were collected from arterial catheters or by direct venipuncture. Whole blood was drawn first into a 2.5-mL plastic syringe without any anticoagulant. The first drop of blood was immediately applied to the test strip to obtain the CoaguChek INR. Subsequently, 1.8 mL of blood was dispensed and mixed in a collection tube containing sodium citrate for assessing the laboratory INR.

### Statistical analysis

Results are expressed as mean (±standard deviation: SD) for normally distributed continuous variables and median (interquartile range: IQR) for variables with a skewed distribution. Categorical variables are reported as counts and percentages. Statistical methods recommended by the CLSI C28-A3 document were used to define the RIs [9]. Normality was evaluated by histograms for the variable and the one-sample Shapiro-Wilk test. The presence of outliers was determined using Dixon’s test [9]. The RIs for INR were defined by nonparametric 95th percentile intervals [9]. Correlation between variables was evaluated by Spearman’s correlation coefficient. The Mann-Whitney U test and the Kruskal-Wallis test or chi-square test were used as appropriate. Simple and multivariate regression analyses were employed to evaluate the influence of gender, gestational age, birth weight, nutrition, serum bilirubin and hematocrit value on INR. The Statistical Package for Social Sciences (SPSS version 18, Tokyo, Japan) for Windows was used to manage and analyze the data. A P-value of less than 0.05 was considered to be statistically significant.

## Results

### INR reference intervals

**Figure 1:**
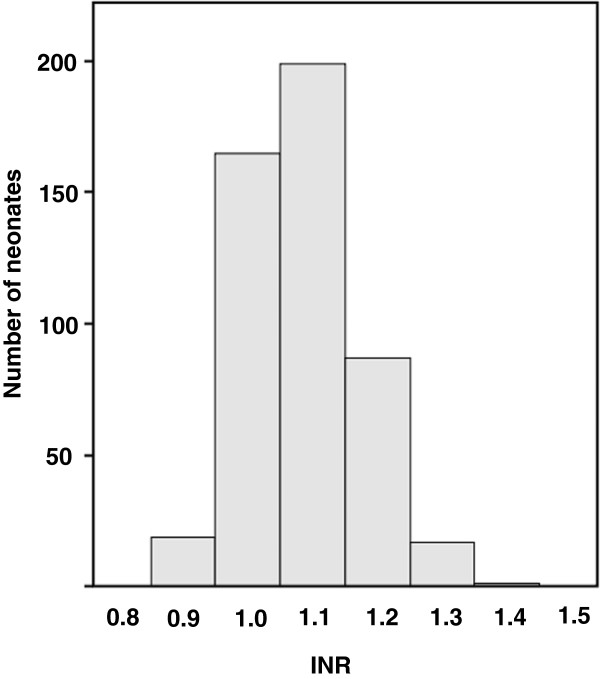
Histogram of international normalized ratios (INRs) measured by CoaguChek XS in 488 healthy term neonates.

This study included 498 healthy term neonates. INR values were measurable in 488 out of all of the enrolled neonates. The sample size was in accordance with the rigorous CLSI guidelines for determining laboratory RIs, which recommend a minimum of 120 subjects for determination of a 95th percentile clinical reference range [9]. Demographic characteristics and laboratory findings of the study population are shown in Table 1. There were no outliers in the INR data. The histogram of INRs of 488 healthy term neonates is shown in Figure 1. It seemed to show an approximately normal distribution, but the Shapiro-Wilk test did not indicate that the distribution of INRs was normal. Accordingly, a non-parametric approach was employed to construct the RIs of INRs. As a result, the median INR was 1.10, and the reference interval (RI) expressed by the 95th percentile interval was between 0.90 and 1.30. A substantial discrepancy existed between the INRs of male and female neonates (p = 0.049), and the gender-specific RIs are shown in Table 2.

**Table 1:** Demographic data of the study population by gender

	**Total**	**Male**	**Female**
n	488	243	245
Gestational age , wks	39.4 (38.6-40.2)	39.4 (38.6-40.3)	39.4 (38.6-40.3)
Birth weight, g	3050 (2806-3294)	3096 (2844-3348)	3000 (2753-3247)
Vaginal delivery, n (%)	395 (81)	201 (83)	194 (79)
Breastfeeding only, n (%)	311 (64)	162 (67)	149 (61)
Serum total bilirubin level, mg/dL	11.3 ± 2.8	11.7 ± 2.9	11.0 ± 2.8
Hemotocrit level, %	52.0 ± 5.8	52.0 ± 6.0	52.1 ± 5.6

Values are presented as median (interquartile rang) or mean ± standard deviation (SD) unless otherwise indicated.

**Table 2:** Reference intervals of INRs in healthy term neonates

**Gender**	**n**	**Median**	**2.5th percentile**	**97.5th percentile**
All	488	1.10	0.90	1.30
Male	243	1.10	0.90	1.30
Female	245	1.10	0.90	1.24

INRs, international normalized ratios.

### Correlation factors

To analyze the influences of the demographic characteristics and laboratory findings on INR, simple linear regression was performed with INR as the dependent variable, and gestational age, birth weight, mode of delivery, nutrition, serum total bilirubin value and hematocrit value as independent variables. We observed negative correlations between INR and gestational age (r = -0.17, p < 0.001) and birth weight (r = -0.20, p < 0.001). INR was significantly correlated with hematocrit value (r = 0.37, p < 0.001) and serum bilirubin value (r = 0.15, p = 0.001). As for the other independent variables, there was no significant correlation between INR and each variable. Next, multiple linear regression was performed in order to clarify the influence of the independent variables on the INR. We thought that this analysis is reliable because the residuals were normally distributed while the INRs were not normally distributed in the Shapiro-Wilk test. As a result, gender significantly influenced the INR value. Inverse correlations were observed between INR and gestational age and birth weight. Conversely, there was a significant positive correlation between INR and hematocrit value. The other independent variables including serum bilirubin value had no significant correlation with INR. The regression coefficients in the multiple linear regression model are shown in Table 3.

**Table 3:** Multiple linear regression model showing the association of INR with predictors

**Factor**	**Regression coefficient**	**P value**
Male gender	0.111	0.006
Gestational age	-0.170	<0.001
Birth weight	-0.147	0.001
Vaginal delivery	0.055	0.203
Breastfeeding only	0.006	0.883
Serum total bilirubin level	0.010	0.814
Hematocrit level	0.387	<0.001

INR, international normalized ratio.

### CoaguChek XS utility

There were 10 failures (failure rate 2%). Failures seemed to be independent of neonate characteristics. In all instances, an INR result could not be obtained due to insufficient sample volume, because the neonate struggled when his heel was pricked and the blood could not be applied to the test strip correctly.

### Accuracy of CoaguChek XS

**Figure 2:**
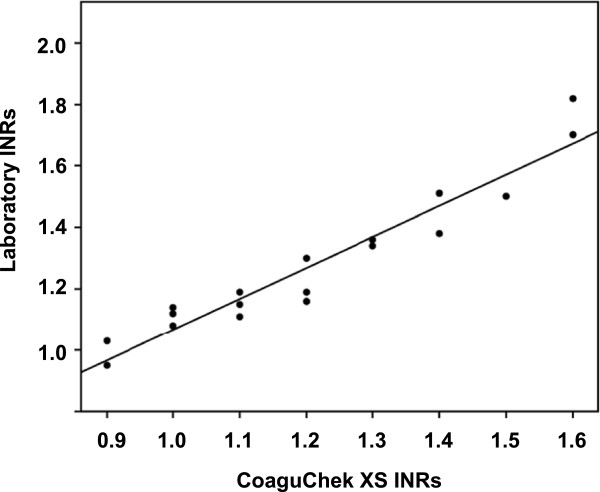
**Relationship between INR values obtained by CoaguChek XS and INR values measured in the laboratory.** n = 18, r = 0.967, p < 0.001.

During the study period, arterial or venous blood samples of 18 patients admitted to the NICU (median gestational age, 38.1 weeks; median birth weight, 2829 g; median age at blood sampling, 3 days after birth) were taken to determine INR values using the CoaguChek XS and those in the central laboratory as the reference method. Figure 2 illustrates the correlation between INRs obtained using the CoaguChek XS and the laboratory method. The overall Spearman correlation coefficient was 0.967 (p < 0.001). In addition, individual differences between laboratory INR and CoaguChek INR values were less than 0.1 in 11 cases (61%) and less than 0.15 in 17 cases (94%). These results demonstrate the CoaguChek XS meter to be accurate for use in neonates.

## Discussion

There has been no previous study on INR in healthy full-term neonates as measured by CoaguChek XS. We successfully established the RIs of CoaguChek INR in this population. According to the manufacturer’s report, the normal range for an INR done with a CoaguChek XS is between 0.9 and 1.1. The RI for INR in neonates in our study was not so different from that in adults. It is known that classic PT is high on the first and second days of life but normalizes by the fifth day [10]. Oral vitamin K administration after birth might contribute to the result.

There was a statistically significant difference in INR values between male and female neonates, with the male group having a significantly higher INR value. Some previous studies reported a trend toward enhanced coagulation in females compared with males; females have a higher rate of fibrin formation, and increased levels of fibrinogen, factor VII, factor VIII, and factor IX and a decreased level of protein C are associated with increased coagulation activation [11,12]. However, these studies were conducted in an adult population. There has been no study investigating gender differences in neonatal coagulation. In our study, the RIs were close between male and female neonates, although they were statistically significant; the gender difference might be irrelevant to detecting prophylactic failure in clinical fields. As for the gender difference of VKDB incidence, a nationwide survey in Japan demonstrated a high incidence of males with VKDB [13]. Bhanchet et al. reported that the ratio of males to females was 1.7 for VKDB [14]. Endogenous hormones may play a role in sex-related differences, but the exact mechanism is unknown.

We observed negative correlations between INR and gestational age and birth weight. Previous studies showed a significant relationship between PT and birth weight as well as gestational age [15]. Our observations clearly confirmed these findings. The hemostatic system evolves gradually throughout gestation and early infancy [16,17]. A previous observation showed that in the healthy human fetus, both PT and INR decreased gradually during gestation, which was clearly related to a significant increase in coagulation factors including vitamin K-dependent proteins [18]. In our study, the INR value decreased during gestation even in term neonates. It is unknown if the pattern of coagulation system differs according to the gestational age among term neonates.

This study revealed that there was a wide range of hematocrit values (37–71%), and we observed a significant correlation between INR and hematocrit value. It is generally thought that the hematocrit level affects the PT measurement as anemia falsely depresses and polycythemia falsely elevates coagulometer readings of INR levels. The classic PT measured in citrate plasma is influenced by the hematocrit due to the dilutional effect of plasma [19,20]. As for INRs determined by CoaguChek XS, there is no influence of citrate and the clotting time in this device depends primarily on the concentration of clotting factors including the endogenous calcium ion concentration in the blood [21]. The hematocrit might also influence the displayed clotting time by the velocity of the blood streaming into the reaction zone of the test strip, or the rate of dissolution of the dried reagents in the blood, or the concentration of reagent dissolved in the blood. It is not known if factors influencing blood viscosity, e.g., fibrinogen, have an effect on the clotting time measured by the CoaguChek XS. According to the manufacturer’s report, hematocrit values ranging between 0.25 and 0.55 do not significantly affect the test results. In our study, hematocrit values were above 0.55 in 27% of the neonates. Polycythemia is a relatively common disorder in neonates. The accuracy of CoaguChek INRs in blood samples with high hematocrit values is an important issue to study further.

In this study, the CoaguChek XS was operated by four neonatologists. All study personnel indicated that they would support the introduction of this device into clinical practice when they were asked about ease of use. The failure rate of measuring INRs by the CoaguChek XS was 2% in our study. A previous study in a pediatric population demonstrated that failure rates were 2% with CoaguChek XS and 4% in the laboratory test [22]. One limitation of this study is that CoaguChek XS INR was measured by a small group of trained neonatologists. CoaguChek XS has been designed especially for use by non-health professionals, so anyone can perform the test. However, if the subject is a neonate, CoaguChek XS INR measured by less well-trained operators might be less reliable. The reliability of CoaguChek INR as measured by a wider group of operators should be verified.

We compared the INR values determined using the CoaguChek XS POC assay with those obtained using a standard laboratory assay to assess the accuracy of the CoaguChek device in neonates. The INR value by CoaguChek XS was closely correlated with the laboratory INR value (r = 0.967). The degree of correlation was the same as those in other studies performed in adults (r = 0.91–0.95) [23,24] and children (r = 0.96–0.97) [8,25].

One strength of the present study was a large sample size compared to previous studies concerned with the coagulation system that included INR data from a very small number of neonates. Another strength was that the sample was composed entirely of healthy individuals, excluding the study on the accuracy of CoaguChek XS. This stands in contrast to previous studies, which mostly included preterm, low birth weight, or morbid neonates.

On the other hand, one limitation of our study is that we did not compare the INR values, determined with the CoaguChek device, with other markers of vitamin K deficiency. The reliability of a coagulation screening test to detect prophylactic failures might be warranted by discovering the hemorrhagic tendency of healthy neonates by chance. Prothrombin time becomes prolonged only when the prothrombin concentration drops below 50% of normal [26]. Therefore, while the PT is appropriate for diagnosis of overt vitamin K deficiency, it might not be useful for detecting subclinical deficiency. Proteins induced by vitamin K absence (PIVKA-II) could be early markers of vitamin K deficiency because they are detectable before there are alterations in other coagulation tests [27]. Normotest is also used as a marker of vitamin K deficiency. Infants with VKDB usually display less than 10% coagulation activity by Normotest, and apparently healthy infants having less than 20% are considered to be in a latent hemorrhagic state [13]. However, these markers need to be measured in a central laboratory. Shortening the delay in obtaining coagulation test results from a central laboratory is one of the critical issues to efficiently prevent VKDB. More research by comparison with PIVKA-II or Normotest is needed before POC measurement of INR can be recommended for neonatal care. Another limitation is the time of INR examination. Because the majority of VKDB infants had a history of prophylactic vitamin K administration [2], INR examination at 4 days after birth might be too early to detect prophylactic failure or incomplete prophylaxis because INR might still be in the normal range from recent vitamin K administration. A follow-up test might be warranted.

## Conclusion

CoaguChek XS appears to be a valid instrument for INR monitoring even in neonates. In this study, we reported the RIs (0.90–1.30) for CoaguChek XS INR in healthy term neonates. In case an INR value exceeds 1.30, frequent (daily or weekly) oral vitamin K administration and follow-up CoaguChek XS INR evaluations should be recommended. If prophylaxis is not effective, detailed investigations (e.g., hemostatic profile tested using venous access blood samples and liver function test to determine total and direct bilirubin levels) are required to diagnose predisposing diseases.

## Abbreviations

RI: Reference interval; INR: International normalized ratio; VKDB: Vitamin K deficiency bleeding; POC: Point-of-care; PT: Prothrombin time; NICU: Neonatal intensive care unit; ISI: International sensitivity index; SD: Standard deviation; IQR: Interquartile range; PIVKA: Proteins induced by vitamin K absence.

## Competing interests

The authors report no potential competing interest.

## Authors’ contributions

SI conceived the study and was responsible for data collection. SI performed data analysis, interpreted the data, and prepared the first draft of the manuscript. TB, DU, and AO made substantial contributions to acquisition of data. All authors reviewed and contributed to revisions of the manuscript. All authors read and approved the final manuscript.

## Pre-publication history

The pre-publication history for this paper can be accessed here:

http://www.biomedcentral.com/1471-2431/14/179/prepub
